# Interleukin-22 and Its Correlation with Disease Activity in Plaque Psoriasis

**DOI:** 10.1007/s00005-018-0527-5

**Published:** 2018-10-05

**Authors:** Bartłomiej Wawrzycki, Aldona Pietrzak, Ewelina Grywalska, Dorota Krasowska, Grażyna Chodorowska, Jacek Roliński

**Affiliations:** 1 Department of Dermatology, Venereology and Pediatric Dermatology, Medical University of Lublin, Lublin, Poland; 2 Department of Clinical Immunology and Immunotherapy, Medical University of Lublin, Chodzki 4a, 20-093 Lublin, Poland

**Keywords:** Immune system, IL-22, Psoriasis, T-cell culture

## Abstract

Psoriasis is a chronic debilitating skin disease with an estimated prevalence reaching 2% of the worldwide population. Psoriatic disease is driven by a network of complicated reciprocal interactions among innate and adaptive mechanisms of immune system with structural components of the skin. Interleukin (IL)-22 mediates keratinocyte proliferation and epidermal hyperplasia, inhibits terminal differentiation of keratinocytes, and induces the production of antimicrobial proteins. The aim of this study was the assessment of IL-22 levels and its correlation with disease activity in plaque psoriasis. The study group included 64 patients with mild, moderate and severe psoriasis. Control group was composed of 24 sex- and age-matched healthy volunteers. IL-22 concentration was assessed in supernatants of T-cell cultures as well as in the plasma of study and control group with the use of ELISA method. Statistical analysis showed that concentration of IL-22 in cultures exposed to staphylococcal enterotoxin B was significantly higher than in control samples (*p* = 0.005) and cultures treated with IL-12 (*p* = 0.005). Patients with psoriasis presented significantly higher concentrations of IL-22 than healthy individuals (*p* = 0.0000001). In conclusion, IL-22 may collaborate with other soluble factors and cells together forming inflammatory circuits that otherwise exist as constitutive or inducible pathways in normal skin and become pathologically amplificated in psoriasis. Targeting IL-22 may be promising as a potential therapeutic for plaque psoriasis.

## Introduction

Psoriasis is a chronic, debilitating skin disease with an estimated prevalence reaching 2% of the worldwide population (Boehncke 2015). Psoriatic disease is driven by a network of complicated reciprocal interactions among innate and adaptive mechanisms of immune system with structural components of the skin (Park and Kupper 2015; Popa et al. 2016).

Currently, psoriasis is believed to be a prototypical autoimmune skin disorder with a central role of interleukin 23/T-helper 17 (IL-23/Th17) axis (Lynde et al. 2014; Martin et al. 2013).

IL-22 is a member of IL-10 family (along with IL-10, IL-19, IL-20, IL-24 and IL-26) which mediates its effects via the IL-22 receptor (IL-22R) complex which is expressed by non-hematopoietic cell lineages of skin, intestine, lung, liver, pancreas and kidney (Dudakov et al. 2015).

IL-22 is produced by various cells of the lymphoid lineage, belonging to both the adaptive and innate immune system: αβ T-cells, γδ T-cells, natural killer T-cells and innate lymphoid cells (Dudakov et al. 2015; Jia and Wu 2014; Wolk et al. 2010). Recent research indicates that IL-22 might be also released by cells of other than lymphoid origin. Best known are human dermal mastocytes (Mashiko et al. 2015) that recently emerged as a predominant source of IL-22 in atopic dermatitis and psoriasis. In certain circumstances of acute tissue injury or bacterial invasion, it might promote survival by stimulating epithelial cell regeneration and production of antimicrobial peptides (Dudakov et al. 2015). On the other hand, chronic states of IL-22 over-expression may reveal its pathologic effects (Dudakov et al. 2015).

Although IL-22R is expressed on dermal fibroblasts, endothelial cells and melanocytes, the keratinocytes are the main target for the IL-22 in the skin (Wolk et al. 2006). Among the most striking effects of IL-22 are upregulation of the expression of genes responsible for antimicrobial defense, cellular differentiation, and mobility in keratinocytes (Sabat and Wolk 2011; Wolk et al. 2006, 2009) implicating a possible important role in inflammatory diseases with marked epidermal acanthosis, such as psoriasis.

The aim of this study was the assessment of IL-22 levels and its correlation with disease activity in plaque psoriasis.

## Materials and Methods

### Participants

The study group included 64 patients, i.e., 9 patients (14.6%) with mild, 26 (40.62%) with moderate and 29 (45.31%) with severe psoriasis (32 women and 32 men), with age between 40 and 78 years (mean 56.7 years). All patients were diagnosed with psoriasis at the Department of Dermatology, Venereology and Pediatric Dermatology, Medical University of Lublin, of a few to several years earlier (minimum 1 year, maximum 8 years), and typically experienced 2–3 recurrences per year. Clinical severity of psoriasis was assessed based on PASI scores, ranging between 4.5 and 36.8 (mean 13.9). Control group comprised 24 sex- and age-matched healthy volunteers. During the study and within the preceding month, none of the patients and controls showed any signs of infection or took any immunomodulating agents. Individuals with a history of other autoimmune, neoplastic and allergic diseases were excluded from the study.

### Ethics

This study was approved by the Ethics Committee of the Medical University of Lublin (decision no. KE-0254/227/2010). Written informed consent was obtained from all patients with respect to the use of their blood for scientific purposes.

### Material Collection

Peripheral blood samples were collected into heparinized tubes (10 ml) and immediately processed. Peripheral blood samples collected into EDTA tubes (5 ml) were used for plasma separation. Plasma samples were stored at − 70 °C until the time of analysis. All laboratory procedures were performed at the Department of Clinical Immunology and Immunotherapy, Medical University of Lublin.

### Isolation of Mononuclear Cells

Peripheral blood collected into heparinized tubes was diluted with 0.9% buffered saline (PBS) without calcium (Ca^2+^) and magnesium (Mg^2+^) (Biochrome AG, Germany) in 1:1 ratio. The diluted material was built up with 3 mL of Gradisol L (specific gravity 1.077 g/ml; Aqua Medica, Poland), and centrifuged in a density gradient at 700×*g* for 20 min. The obtained fraction of peripheral blood mononuclear cells (PBMCs) was collected with Pasteur pipettes and washed twice in PBS without Ca^2+^ and Mg^2+^ for 5 min. Subsequently, the cells were suspended in 1 ml of PBS without Ca^2+^ and Mg^2+^, and either counted in the Neubauer chamber or tested for viability with trypan blue solution (0.4% Trypan Blue Solution, Sigma-Aldrich, Germany). Viability below 98% disqualified the cells from further analyses.

### Determination of IL-22 Concentration in Supernatants of T-Cell Culture Derived from Patients with Psoriasis

T-cells were isolated from the PBMCs of patients, using anti-T CD3^+^ antibodies coating MicroBeads (Miltenyi Biotec, USA), which enabled a positive selection of T-cells. T-cells were cultured under standard conditions (37 °C, 5% CO_2_) in RPMI 1640 medium (PAA Laboratories, Austria), enriched with 2% human albumin (Baxter, USA) and supplemented with antibiotics: penicillin (100 IU/ml), streptomycin (50 µg/ml), neomycin (100 µg/ml), (Germany). Equal amounts (1 × 10^6^) of the T-cells were treated according to the following combinations for 72 h: (1) control—unstimulated T-cells, (2) T-cells stimulated with 25 ng/ml phorbol esters (PMA: phorbol 12-myristate 13-acetate; Sigma, Germany), (3) T-cells stimulated with 10 ng/ml bacterial polysaccharides, (lipopolysaccharides from *Escherichia coli* 026:B6; Sigma-Aldrich, USA), (4) T-cells stimulated with 5 ng/ml IL-12 (IL-12 human, Sigma-Aldrich, USA), and (5) T-cells stimulated with 100 ng/ml staphylococcal enterotoxin B (SEB: Staphylococcal enterotoxin B; Sigma-Aldrich, USA). Aspirated cell culture supernatants were centrifuged at 300×*g* for 10 min, collected to Eppendorf tubes (3 × 200 µl), frozen and stored at − 70 °C until analysis. A commercial enzyme-linked immunosorbent assay (ELISA) kit Quantikine Human IL-22 Immunoassay (R&D Systems, USA) with the sensitivity of 2.7 pg/ml was used for a quantitative determination of human IL-22 in plasma samples. The protocols which followed were in accordance with the manufacturer’s recommendations. The ELISA Reader VictorTM3 (PerkinElmer, USA) was used.

### Determination of IL-22 Concentration in the Plasma

Five milliliters of peripheral blood samples collected into EDTA tubes were used for plasma separation. Plasma samples were stored at − 70 °C until the time of analysis. A commercial ELISA kit was used as described above.

### Statistical Analysis

Normal distribution of continuous variables was tested using the Shapiro–Wilk test. Statistical characteristics of the continuous variables were presented as medians, minimum and maximum values, as well as arithmetic means and their standard deviations (SD). The Student’s *t* test was used for independent variables, and the Mann–Whitney *U* test was used for intergroup comparisons. The power and direction of relationships between pairs of continuous variables were determined on the basis of the values of Spearman’s coefficient of rank correlation (*R*). All the calculations were carried out with Statistica 10 (StatSoft^®^, USA) package, with the level of significance set at *p* < 0.05.

## Results

### Comparison of Plasma Concentrations of IL-22 in Patients with Psoriasis and Controls

Statistical analysis revealed that patients with psoriasis presented significantly higher concentrations of IL-22 than healthy individuals (*p* = 0.0000001). Mean concentration of IL-22 in psoriatic patients was 39.56 ± 81.77 pg/ml (range 0.0–492.34 pg/ml), and mean concentration of this cytokine in the controls amounted to 1.70 ± 2.96 pg/ml (range 0.0–11.146 pg/ml).

We examined a relation between the plasma concentration of IL-22 and the severity of psoriasis (PASI score). Statistical analysis revealed a significant positive correlation between PASI scores and plasma concentrations of IL-22 (*R* = 0.57, *p* = 0.000042).

### Concentration of IL-22 in Lymphocyte Culture Supernatants

Statistical analysis documented significant differences in IL-22 concentrations in cell culture supernatants from various experimental variants (*p* = 0.0003). Post hoc analysis showed that concentration of IL-22 in cultures exposed to SEB was significantly higher than in control samples (*p* = 0.005) and cultures treated with IL-12 (*p* = 0.005). The remaining experimental variants did not differ significantly in terms of their IL-22 concentrations (*p* > 0.05; Table 1).

**Table 1:** Mean IL-22 (pg/ml) concentration in lymphocyte culture supernatants and control samples

Group	Mean	Median	Lower quartile	Upper quartile	SD
IL-12	0.00	0.00	0.00	0.00	0.00
SEB	1090.66	1104.41	1104.41	1104.41	30.75
PMA	3.07	2.15	1.86	2.53	2.22
Control samples	0.00	0.00	0.00	0.00	0.00

*H* = 18.51; *p* = 0.0003

*IL-12* interleukin 12, *SEB* staphylococcal enterotoxin B, *PMA* phorbol 12-myristate 13-acetate

## Discussion

Although psoriasis is considered to be a prototypical T-cell-dependent disease with aberrant response of activated T-lymphocytes against some hitherto undefined cutaneous antigen, the exact events leading to such activation and nature of the putative autoantigen are still a matter of controversy (Anand et al. 2017; Sticherling 2016).

With regard to memory CD4 T-cells Th1, Th17, and Th22 lymphocytes are capable of secreting IL-22 (Dudakov et al. 2015). Traditionally, IL-22 was considered as a Th17 cytokine (Sabat and Wolk 2011). Several studies from the past few years showed that the number of lymphocytes secreting uniquely IL-17 or IL-22 may outnumber those releasing both mediators (Duhen et al. 2009; Nograles et al. 2008; Trifari et al. 2009). This new subset of T-helper lymphocytes called Th22 was identified by a secretory capabilities of abundant IL-22 production without concomitant expression of interferon γ, IL-4 and IL-17 (Jia and Wu 2014; Mirshafiey et al. 2015; Perusina Lanfranca et al. 2016). Interestingly, they are equipped in skin homing CCR4 and CCR10 receptors which indicate importance of that cell subset in the skin. Based on current knowledge, production of IL-22 does not seem to be restricted to any particular T-cell lineage (Ahlfors et al. 2014). It depends rather on target tissue microenvironment then cell morphology. Cell commitment to synthesize either of cytokines may also depend on type of stimulus (acute vs. chronic), extension (local or systemic), type of pathogen and affected tissue (Wolk et al. 2010).

In our study, serum levels of IL-22 were elevated in psoriatic patients compared to healthy controls which stays in line with previous reports (Boniface et al. 2007; Lo et al. 2010; Shimauchi et al. 2013; Wolk et al. 2006). The levels of IL-22 correlated with disease severity as measured by Psoriasis Area Severity Index (PASI) similarly to the above-mentioned studies (Boniface et al. 2007; Lo et al. 2010; Shimauchi et al. 2013) which may suggest that IL-22 expression is more than just bystander phenomenon.

First line of the evidence connecting IL-22 to psoriasis pathogenesis comes from genetic studies. IL-22 variant that promotes epithelial barrier defense was found preferentially enriched in a investigated population associated with the onset of disease at an early age (Nikamo et al. 2014; Prans et al. 2013).

Until recently, psoriasis has been believed to be strictly mediated by autoimmune mechanisms. Now it is known that it rather originates from three different biological pathways (adaptive immunity, innate immunity, and skin barrier) (Bergboer et al. 2012).

Knowledge about important role of epidermal barrier function, its integrity and responses to microbial environment allowed to form a hypothesis according to which psoriasis could be considered as a “barrier organ disease” (Mattozzi et al. 2012) analogically to the archetypical member of such disorders—Crohn disease (CD) (Schreiber et al. 2005). It has been known for a long time that patients with CD and their first-degree relatives have increased risk of developing psoriasis and vice versa (Egeberg et al. 2016). Very recently, it was proposed that similar to CD (Øyri et al. 2015), psoriasis could be a result of breakdown of immune tolerance (dysbiosis) to cutaneous bacteria (Fry et al. 2013, 2015). According to the aforementioned theory, genetic abnormalities affecting especially innate immune response facilitate initiation of inflammatory response to cutaneous microbiota (Skroza et al. 2013; Terziroli Beretta-Piccoli et al. 2017; Vlachos et al. 2016; Wu et al. 2012).

In our study, concentration of IL-22 in supernatants from PBMCs was significantly higher in psoriatic patients compared to healthy controls. More importantly, we have found that PBMCs from patients with psoriasis stimulated with SEB secreted significantly higher concentrations of IL-22 than those stimulated with IL-12, or PMA. This is similar to the results from Niebuhr et al. (2010, 2014) implicating altered response to bacterial products in psoriasis.

The best known triggering factors of psoriasis in susceptible individuals are drugs and infections (Fry and Baker 2007). With regard to the latter, streptococcal throat infections are the most commonly inducing or aggravating factor in a subset of patients who suffer from an acute form of the disease—guttate psoriasis (Sigurdardottir et al. 2013; Thorleifsdottir et al. 2016). Conversely, little is known about the potential impact of cutaneous microflora especially in chronic stable psoriasis which constituted 100% of our investigated group.

In opposition to atopic dermatitis (Biedermann et al. 2015; Nakatsuji et al. 2016; Ong and Leung 2010; Totté et al. 2016a) where chronic colonization with *Staphylococcus aureus* is a major factor driving chronic inflammatory state and disease flares, data concerning its role in psoriasis are sparse. Few studies which investigated this subject revealed increased skin colonization with *S. aureus* (Tomi et al. 2005; Totté et al. 2016b)—in more than 50% of psoriatics (reaching 100% of those with erythroderma) vs. 12% of healthy controls. Moreover, disease activity was significantly correlated with toxogenic strains (Balci et al. 2009; Tomi et al. 2005).

IL-22 was found to be crucial in cutaneous immunity against staphylococcal infection first through the induction of adenosine monophosphates (AMPs) (Mulcahy et al. 2016; Wolk et al. 2006) synthesis and second by influencing T-cell and neutrophil chemotaxis (Chan et al. 2015). Production of IL-22 in these circumstances could be in part attributable to population of skin γδ T-cells (Malhotra et al. 2016). IL-22, mainly of γδ T-cell origin, was found to be necessary to sustain the psoriasiform skin inflammation in the mouse model triggered by TLR7/8 agonist—imiquimod (Van Belle et al. 2012). Those γδ cells formed dermal reservoir of long-lived memory lymphocytes, persisting long after initial stimulation (Hartwig et al. 2015). From a practical point of view, this could explain psoriasis chronicity and relapses. It seems that IL-22 along with innate immune memory contributes to localized (cutaneous) host defense against *Staphylococci* (Muszer et al. 2015; Nowicka et al. 2018a, b).

Hence, IL-22 may collaborate with other soluble factors and cells together forming inflammatory circuits that otherwise exist as constitutive or inducible pathways in normal skin and become pathologically amplificated in psoriasis (Boehncke 2015). Targeting IL-22 may have promise as a potential therapeutic agent for plaque psoriasis.
